# ITAM Signaling by Vav Family Rho Guanine Nucleotide Exchange Factors Regulates Interstitial Transit Rates of Neutrophils *In Vivo*

**DOI:** 10.1371/journal.pone.0004652

**Published:** 2009-02-27

**Authors:** Daniel B. Graham, Bernd H. Zinselmeyer, Francesca Mascarenhas, Ryan Delgado, Mark J. Miller, Wojciech Swat

**Affiliations:** Department of Pathology and Immunology, Washington University School of Medicine, St Louis, Missouri, United States of America; New York University School of Medicine, United States of America

## Abstract

**Background:**

In response to infection, neutrophils are quickly recruited from the blood into inflamed tissues. The interstitial migration of neutrophils is crucial for the efficient capture and control of rapidly proliferating microbes before microbial growth can overwhelm the host's defenses. However, the molecular mechanisms that regulate interstitial migration are incompletely understood.

**Methodology/Principal Findings:**

Here, we use two-photon microscopy (2PM) to study discrete steps of neutrophil responses during subcutaneous infection with bacteria. Our study demonstrates that signals emanating from ITAM-containing receptors mediated by Vav family Rho GEFs control the velocity, but not the directionality, of neutrophil migration towards sites of bacterial infection.

**Conclusions/Significance:**

Here we show that during neutrophil migration towards sites of bacterial infection, signals emanating from ITAM-containing receptors specifically control interstitial neutrophil velocity.

## Introduction

Neutrophils provide a first line of defense for controlling microbial proliferation in the host, a function critically dependent on the rates of interstitial migration to foci of infection [1], [2], [3]. The recruitment of neutrophils is a complex multi-step process that involves several cell signaling pathways [4]. Neutrophil extravasation begins with selectin-mediated rolling in vessels, followed by integrin dependent firm adhesion, and finally GPCR-mediated diapedesis across the endothelium. After entering peripheral tissues, neutrophils assume amoeboid motility and migrate along chemokine gradients towards sites of infection. Although neutrophil migration has been extensively studied, and the mechanism of extravasation from the blood into infected tissue is relatively well understood [4], the intracellular signals that regulate neutrophil migration through interstitial tissue are not fully elucidated. A particular issue is the exact contribution of signals emanating from G-protein coupled receptors (GPCR), triggered by chemokines, and signals from immunoreceptor tyrosine based activation motif (ITAM)-associated integrin receptors, mediated by interactions with the extracellular matrix [5], [6]. In this context, we have recently shown that, in neutrophils, a combined deficiency in Vav family guanine nucleotide exchange factors (GEF) [7] disrupts integrin-mediated ITAM signaling but permits intact GPCR-mediated activation of downstream effectors [8], [9]. Here, we investigated the role of Vav family Rho GEFs in neutrophil interstitial migration *in vivo* by two-photon microscopy (2PM) [10] using a novel approach that allows direct side-by-side analyses of neutrophils derived from wild type (WT) and Vav^NULL^ mice [7].

## Results and Discussion

**Figure 1:**
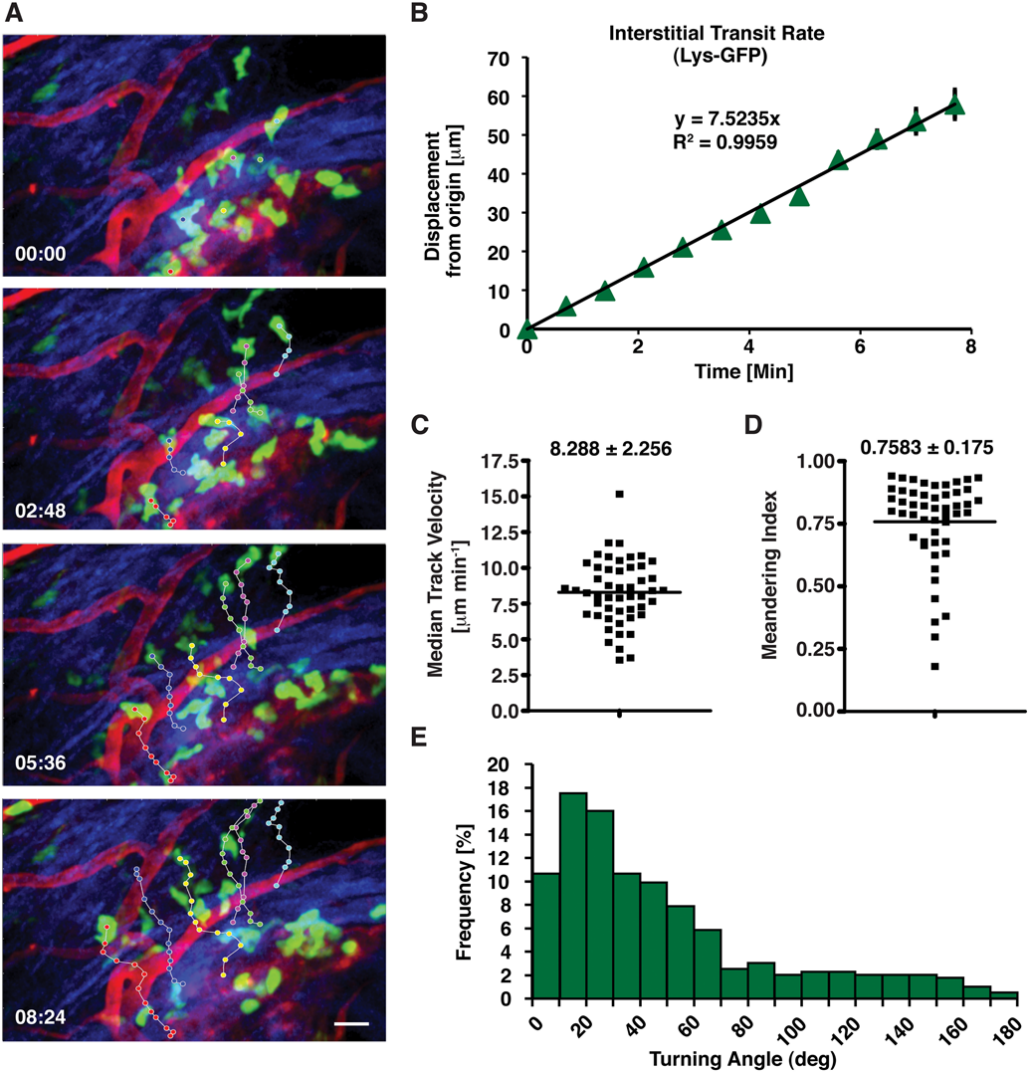
Neutrophils display highly directional amoeboid motility in infected footpad and phalanges. Intravital imaging was performed in the footpad of LysM-eGFP mice 20 min after infection with *Listeria monocytogenes* (LM). (A) Image sequences illustrate typical neutrophil migration patterns (multi-colored dots). Cell centroids were tracked at successive 42 sec intervals using PicViewer [10], [26]. Blood vessels (red) were labeled by i.v. injection of 1 mg dextran-tetramethylrhodamine 2,000,000 MW 5 min prior to imaging. Neutrophils (eGFP) appear green and collagen fibers in the connective tissue (second harmonic generation signal) appear blue. Scale bar = 20 µm. (B) The plot shows the mean displacement of 50 neutrophil tracks (containing a minimum of 9 and a maximum of 12 time points) from five separate experiments. Error bars represent standard error of the mean (SEM). Neutrophil displacement over time is linear (R^2^ = 0.9959), and slope of the regression line (7.5235 µm/min) yields the interstitial transit rate, a convenient measure of how rapidly the cells migrate through the specific local tissue environment. (C) The Plot shows the median track velocity (8.288 µm/min+/−SD 2.256 µm/min) of the tracks in (B). (D) The meandering index calculated for each track was plotted. This value was obtained by dividing the displacement from the origin (after 8 time steps or 5.6 min) by the track length. The median meandering index equals 0.7583+/−SD of 0.175, which indicates migration has a strong directional bias. (E) The turning angles of neutrophil tracks were determined by calculating the angle change between vectors constructed from each time point (42 sec intervals). Angles are given in absolute values calculated by using the arc sine of the dot product of the two normalized vectors.

To establish a baseline for endogenous neutrophil responses, we imaged LysM-GFP mice [11], in which neutrophils are brightly fluorescent by 2PM [12], [13]. The footpad and phalanges were injected subcutaneously (s.c.). with *Listeria monocytogenes* (LM), and within 30 minutes, large numbers of neutrophils were found adhering to the vessel endothelium (Fig. 1 and Movie S1). By 60 minutes, many neutrophils had extravasated and were migrating through the connective tissue (Fig. 1 and Movie S1). We analyzed several neutrophil motility parameters including displacement from origin over time, cell velocity, meandering, and changes in cell shape. Mean neutrophil displacement over time was distinctly linear (Fig. 1B), therefore migration was expressed as an “interstitial transit rate” (µm/min) in contrast to the lymphocyte motility coefficient, which is expressed as displacement squared over time [14]. In time-lapse video recordings, neutrophils showed a strong directional bias in their migration, consistent with chemotaxis (Movie S1). LysM-eGFP neutrophils migrated with a median velocity of ∼8 µm/min (Fig. 1C), which is slightly lower than the velocity of swarming neutrophils in lymph nodes [12], but similar to values reported for neutrophils responding to infection in the ear [13] and adoptively transferred neutrophils recruited to the footpad [10].

**Figure 2:**
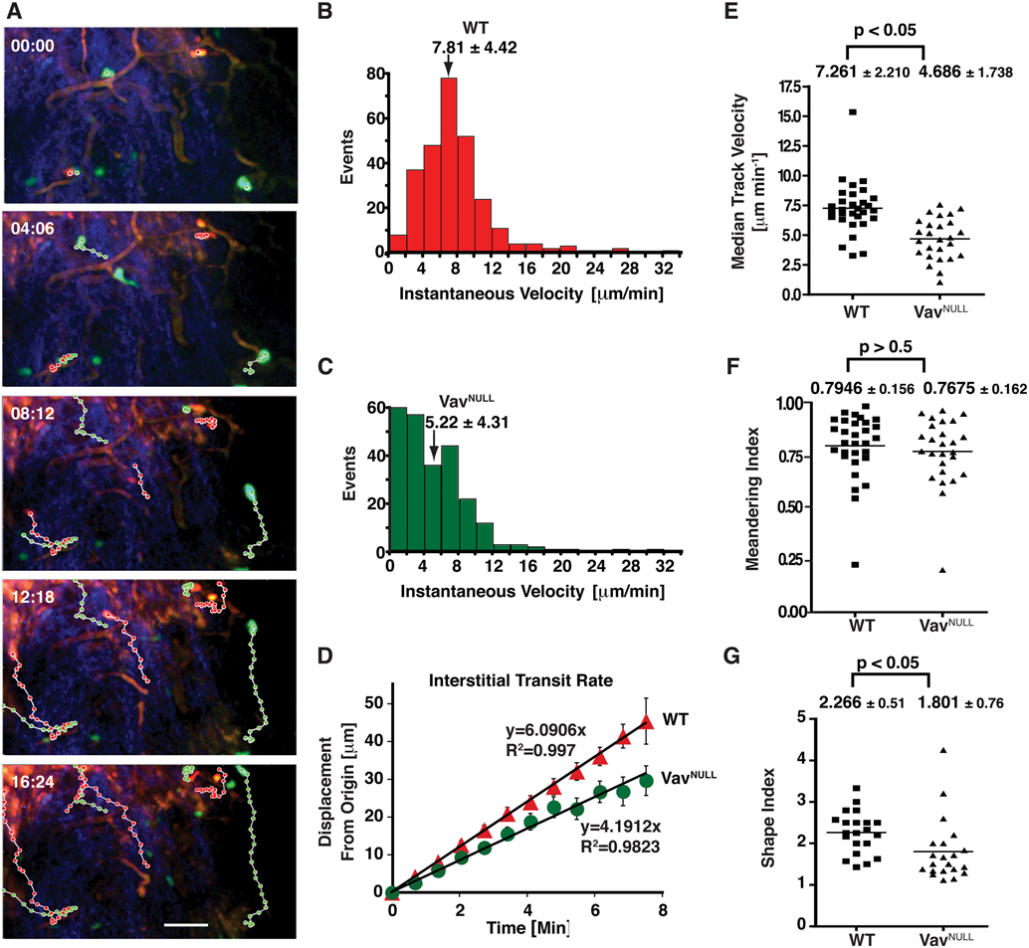
Vav GEFs control the interstitial transit rate of neutrophils without affecting directionality. Adoptively transferred Vav^NULL^ (green; CFSE labeled) and WT (red; CMTPX-labeled) neutrophils were imaged by time-lapse 2PM. Intravital imaging was performed in the footpad of WT mice 20 min after infection with LM. Data compiled from four separate experiments is shown. In 2 experiments, dye staining was reversed to exclude potential dye-induced changes in cell motility. (A) Image sequences illustrate typical neutrophil migration patterns (green-dots for Vav^NULL^, red-dots for WT neutrophils). Cell centroids were tracked at successive 41 sec intervals using PicViewer. Blood vessels (orange) were labeled by i.v. injection of 1 mg dextran-tetramethylrhodamine 2,000,000 MW 5 min prior to imaging. Neutrophils appear green and reddish respectively and collagen fibers in the connective tissue (second harmonic generation signal) appear blue. Scale bar = 30 µm. (B) The histogram shows the instantaneous velocity distribution of WT neutrophils. The distribution has a bell-shaped symmetrical character with a median velocity of 7.81 µm/min (+/−SD 4.42 µm/min). (C) In contrast to WT neutrophils, the instantaneous velocity distribution of Vav^NULL^ neutrophils is skewed with a median velocity of 5.22 µm/min (+/−SD 4.31 µm/min). (D) The plot shows the mean displacement of 30 WT and 26 Vav^NULL^ neutrophil tracks. Error bars represent standard error of the mean (SEM). Neutrophil displacement over time is linear for wild type (R^2^ = 0.997) and Vav^NULL^ neutrophil tracks (R^2^ = 0.9823). The slope of the regression line yields the interstitial transit rate of 6.09 µm/min for WT neutrophils and 4.19 µm/min for Vav^NULL^ neutrophils. (E) The Plot shows median track velocities of tracks in (B). The average median track velocity is statistically different (p value = 0.0001) between WT neutrophils (7.261 µm/min+/−SD 2.210 µm/min) and Vav^NULL^ neutrophils (4.686 µm/min+/−SD 1.738 µm/min). (F) The meandering index was calculated by dividing the cell's displacement from the origin (after 8 time steps or 5.6 min) by the track length. Meandering values for individual tracks were plotted. No statistical differences (p value = 0.525) were observed between WT neutrophils (0.7946+/−SD of 0.156) and Vav^NULL^ neutrophils (0.7675+/−SD of 0.162). (G) The cell shape index for WT and Vav^NULL^ neutrophils was calculated as the ratio of the cell's length (from the leading edge to uropod) to the cell's width (taken perpendicular to the axis of migration). WT cells had significantly (p value = 0.0245) more elongated shapes (shape index = 2.266+/−SD 0.51) compared to Vav^NULL^ neutrophils (shape index = 1.801+/−SD 0.76). Comparisons were made using CFSE-stained WT and Vav^NULL^ cells.

To quantify the directional component of neutrophil migration we calculated the meandering index (a ratio of displacement from origin to track length) on an individual track-by-track basis. These analyses revealed an average meandering index of >0.76 (Fig. 1D), indicating that neutrophil paths in interstitial tissue are remarkably straight, compared with the more tortuous paths of lymphocytes [14] and neutrophils within lymph nodes [12]. Additionally, the directional component of migration can be determined by measuring the turning angles of neutrophil tracks as determined by calculating the angle change between vectors constructed from each time point at 42 second intervals [15]. The peak distribution of angle changes occurs between 10–20° (Fig. 1E), indicating highly directed migration, which corroborates our measurement of the meandering index (Fig. 1D). During migration, we also noted dramatic changes in neutrophil morphology. Analysis of shape index (length/width) revealed a significant elongation of cell bodies (Fig. 2, and data not shown). Taken together, these data strongly indicate that the mode of motility employed by neutrophils in interstitial tissues is consistent with a previously described model of amoeboid motility [16], characterized by high speed adhesion-independent movement associated with a low degree of cytoskeletal organization and a lack of discrete focal contacts.

Neutrophil migration requires crosstalk between G-protein coupled receptor signals generated by chemotactic cues and ITAM signals mediated by integrin receptor interactions with the extracellular matrix [4], [17]. While integrins are not essential for neutrophil motility in 3D collagen matrices or for dendritic cell migration in the lymphatics [18], the contribution of ITAM signals to neutrophil migration in inflamed connective tissues remains to be elucidated. We have previously developed Vav^NULL^ mice as a model in which ITAM signaling is uncoupled from GPCR-induced chemokine signals, and we recently showed that Vav is required for activation of Rac downstream of integrin-mediated ITAM signals, but not GPCR-mediated signals [7], [8], [9]. Indeed, while ITAM pathways utilize Vav GEFs to activate Rac, GPCRs utilize the GEFs P-Rex1 and DOCK2 [19], [20], [21]. Therefore, Vav^NULL^ mice provided us with a unique tool to interrogate the importance of ITAM-dependent signals in neutrophil motility, as Vav^NULL^ neutrophils show a nearly complete block in ITAM signaling, but can still undergo integrin activation via inside-out signaling and undergo chemotaxis in response to chemotactic gradients [8].

To determine if Vav-dependent ITAM signals are required for neutrophil motility, we developed a competitive assay in which Vav^NULL^ neutrophils were analyzed *in vivo* side-by-side with wild type (WT) neutrophils. WT recipient mice were infected with LM, and 2 hours later, equal numbers of fluorescently labeled WT and Vav^NULL^ neutrophils were adoptively transferred. Mice were injected i.v. with high molecular weight rhodamine-dextran to visualize blood vessels and to distinguish neutrophils in the blood from extravasated neutrophils. In response to LM infection, adoptively transferred WT neutrophils showed similar extravasation and migration behaviors as compared to endogenous neutrophils (Fig. 1 and Fig. 2) validating this approach. Although Vav and ITAM signaling have been implicated in firm adhesion [8], [22], our analyses showed that substantial numbers of WT and Vav^NULL^ neutrophils extravasated to interstitial tissues (Fig. 2 and Movie S2). Moreover, Vav^NULL^ neutrophils showed a strong directional bias in their migration, similar to WT cells, suggesting that the chemotactic response induced by LM infection is not disrupted by Vav deficiency (Fig. 2A and Movie S2).

Next, we examined migration of Vav^NULL^ neutrophils by measuring several motility parameters (Fig. 2). The instantaneous velocity values of WT neutrophils were normally distributed with a median velocity of 7.81 µm/min (+/−SD 4.42 µm/min) (Fig. 2B). In marked contrast, the velocity distribution of Vav^NULL^ neutrophils was skewed with many cell velocities <4 µm/min and a median velocity of 5.22 µm/min (+/−SD 4.31 µm/min) (Fig. 2C). Strikingly, Vav^NULL^ neutrophils had significantly reduced interstitial transit rates compared to WT neutrophils (Fig. 2D). Moreover, when compared on a per track basis, Vav^NULL^ neutrophils exhibited a 35% reduction in median velocity (Fig. 2E). While the velocity of Vav^NULL^ neutrophils was substantially impaired, their migration remained highly directional, as measured by the meandering index (Fig. 2F). Both meandering index and turning angle distribution were similar between WT and Vav^NULL^ neutrophils (Fig. 2F and data not shown) as well as in WT Lys-GFP neutrophils (Fig. 1E). Thus, these data show that Vav GEFs play a novel role in regulating the interstitial velocity of neutrophils, but they are not required for maintaining the direction of migration during chemotaxis. To determine if the observed defect in velocity was associated with abnormal cell polarity, we measured the shape index (length/width ratio) of neutrophils during migration. Vav^NULL^ neutrophils were significantly less elongated as compared to WT cells (Fig. 2G), consistent with the requirement for Vav in amoeboid motility.

The observation that Vav^NULL^ neutrophils exhibit defects in velocity *in vivo* contrasts with our observation *in vitro* that Vav^NULL^ neutrophils show no reduction in velocity over two-dimensional substrates in response to chemotactic factors [8]. The present study illustrates that the mechanism of neutrophil migration *in vitro* may differ considerably from migration in the native tissue. In contrast to haptokinetic motility *in vitro*, motility in three dimensions *in vivo* shows a clear requirement for Vav proteins and implicates ITAM signaling in the control of interstitial trafficking. In this context, Vav proteins have previously been shown to play essential roles as antigen and DAP12/FcRγ receptor-proximal phospho-tyrosine adaptors. Thus, along with SLP76, Grb2, and Syk, Vav proteins constitute a critical component of the ITAM signalosome [23], [24], [25]. Our work suggests that the activation of this pathway enhances the interstitial transit rate of neutrophils, and as a consequence, augments antimicrobial responses by permitting neutrophils to rapidly infiltrate sites of infection and eliminate bacteria before their numbers overwhelm the host immune system [2], [8].

## Materials and Methods

### Mice

Mice genetically deficient in Vav1, Vav2, and Vav3 (Vav^NULL^) have been previously described [7]. LysM-GFP mice [11] were a generous gift from Klaus Ley (La Jolla Institute for Allergy and Immunology, La Jolla, CA) who backcrossed the originally generated mice from T. Graf (Albert Einstein College of Medicine, Bronx, NY) to the B6 background. Heterozygous mice were used for imaging and strain-matched B6 mice used as controls. All protocols involving mice were carried out in accordance with institutional guidelines and regulations and were approved by the Institutional Animal Care and Use Committee at Washington University School of Medicine.

### Neutrophil Purification

Bone marrow was extracted from femurs and tibias and red blood cells were lysed in hypotonic saline solution. Cells were washed and resuspended in HBSS (without Ca^2+^ and Mg^2+^). Bone marrow cells were then overlayed on a discontinuous gradient of Percoll in HBSS, which was comprised of two steps: a lower 70% and upper 55% fraction. Neutrophils recovered from between the 55% and 70% fractions were washed and resuspended in PBS. Analysis of neutrophil purity by FACS and morphology demonstrated that neutrophil purity was consistently greater than 90%.

### Imaging *in vivo*

Neutrophil imaging in the footpad of mice was performed as previously described [10]. In brief, 20×10^6^ CFU of *L. monocytogenes* (LM) was injected into the footpad of mice 2 hours prior to i.v. administration of bone marrow neutrophils stained with either CFSE (5 uM) or CMTPX (10 uM) for 40 min at 37°C. Additionally, blood vessels were visualized by retro-orbital injection of dextran tetramethylrhodamine (2,000,000 MW). Immediately following injection of neutrophils and dextran, mice were anesthetized and secured to a warming plate on the microscope stage for imaging of inflammation in the footpad. Time-lapse imaging was performed using a custom-built two-photon imaging system [10]. Each plane represents an image 200 µm by 225 µm (xy dimensions), and 21–61 sequential planes were acquired at 2.5 µm increments in the z-dimension to obtain z-stacks. Z-stacks were acquired every 18–48 seconds during time-lapse recordings. Image reconstruction and multi-dimensional rendering were performed with Imaris (Bitplane), while tracking and measuring aspects of cellular motility were performed with Pic Viewer Software (John Dempster, University of Strathclyde) [10], [26].

### Statistical Analysis

Groups of WT and Vav^NULL^ neutrophils were compared using the Mann-Whitney U-test.

## Supporting Information

Movie S1 Neutrophils display highly directional amoeboid motility in infected footpad and phalanges. Intravital imaging was performed in the footpad path of LysM-eGFP mice 20 min after infection with LM.
(6.62 MB MOV)

Movie S2 Vav GEFs control the interstitial transit rate of neutrophils without affecting directionality. Adoptively transferred VavNULL (green; CFSE-labeled) and WT (red; CMTPX-labeled) neutrophils were imaged by time-lapse 2PM. Intravital imaging was performed in the footpad path of WT mice 20 min after infection with LM.
(6.08 MB MOV)
